# Low-Scaling *GW* Algorithm Applied to Twisted Transition-Metal Dichalcogenide Heterobilayers

**DOI:** 10.1021/acs.jctc.3c01230

**Published:** 2024-02-14

**Authors:** Maximilian Graml, Klaus Zollner, Daniel Hernangómez-Pérez, Paulo E. Faria Junior, Jan Wilhelm

**Affiliations:** † Institute of Theoretical Physics, University of Regensburg, 93053 Regensburg, Germany; ‡ Regensburg Center for Ultrafast Nanoscopy (RUN), University of Regensburg, 93053 Regensburg, Germany; ¶ Department of Molecular Chemistry and Materials Science, Weizmann Institute of Science, Rehovot 7610001, Israel

## Abstract

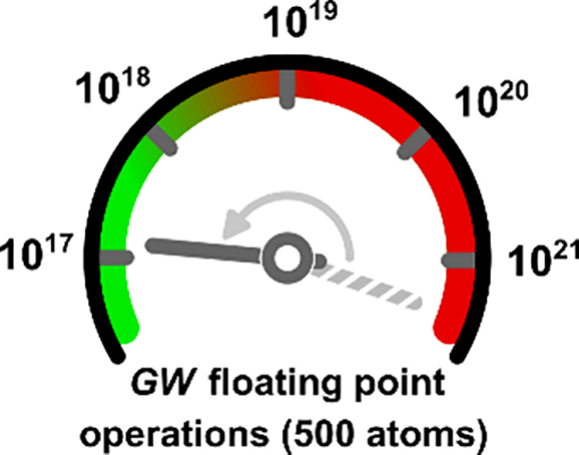

The *GW* method is widely used for calculating the electronic band structure of materials. The high computational cost of *GW* algorithms prohibits their application to many systems of interest. We present a periodic, low-scaling, and highly efficient *GW* algorithm that benefits from the locality of the Gaussian basis and the polarizability. The algorithm enables *G*_0_*W*_0_ calculations on a MoSe_2_/WS_2_ bilayer with 984 atoms per unit cell, in 42 h using 1536 cores. This is 4 orders of magnitude faster than a plane-wave *G*_0_*W*_0_ algorithm, allowing for unprecedented computational studies of electronic excitations at the nanoscale.

## 1 Introduction

Electronic excitations in matter play a pivotal role in various physical phenomena, including light absorption and transport. The characteristics of these excitations are strongly influenced by the host material. Excitons, which are bound electron–hole pairs, exhibit a remarkable and unusually strong electron–hole binding in low-dimensional semiconductors that have emerged in the past decade.^1^ When stacking two atomically thin semiconductors on top of each other, the atomic alignment between the layers can exhibit periodic variations, leading to a new type of in-plane superlattice known as the moiré superlattice. Excitons in moiré structures have gained enormous attention recently^2−12^ thanks to their highly unusual exciton properties which include spatial confinement due to the moiré potential,^2^ interlayer,^5,6^ and intralayer charge transfer.^9^ Furthermore, electronic properties of moiré lattices can be tuned by the band alignment and the twist angle between the layers such that moiré structures hold great promise as an exciting platform for probing electronic and photonic quantum phenomena over the next decade.^12^

Gaining insight into excitons in moiré structures can be achieved through a combination of experiments, theoretical models, and computations. As an example, low-angle MoSe_2_/WS_2_ moiré structures have shown an interesting interplay of intra- and interlayer exciton hybridization because of the nearly degenerate conduction bands of the MoSe_2_ and WS_2_ layers. The conduction band offset and the wave function hybridization between layers, however, are still under debate.^3,7,13−15^ Detailed knowledge about the electronic band structure of the MoSe_2_/WS_2_ moiré bilayer and the implications for exciton formation and binding is thus crucial to resolving this controversy.

In this work, we focus on the *GW* method from many-body perturbation theory,^16−18^ which is an approximation for the electronic self-energy that allows for computing the electronic band structure of a given material. Importantly, *GW* accounts for the nonlocal, frequency-dependent screening of the interaction between electrons, which is crucial in moiré bilayers. The *GW* band structure is then the basis for the description of excitons via the Bethe–Salpeter equation.^17,19^ Currently available plane-wave-based *GW* algorithms are, however, incapable of treating low-angle moiré cells that contain thousands of atoms,^20^ despite their computational scalability to the largest supercomputers.^21−25^ Stochastic *GW* methods^26^ may enable large-scale *GW* calculations; however, it remains uncertain as to whether applying periodic boundary conditions^27^ within the framework of stochastic *GW* can be achieved seamlessly.^28^ To compute the *GW* band structure in large moiré cells, the pristine unit-cell matrix projection (PUMP) has been suggested.^9,20^ PUMP is based on expanding the moiré cell wave functions in terms of the pristine unit-cell wave functions. By construction, PUMP cannot capture the nanometer-scale atomic reconstruction of moiré structures which can dramatically influence their electronic band structure.^4^

The *GW* space–time method^29^ offers a promising route toward large-scale *GW* calculations. This is because the computational scaling is reduced from *O*(*N*_at_^4^*N*_*k*_^2^) for standard *GW* algorithms to *O*(*N*_at_^3^*N*_*k*_) in the *GW* space–time method, where *N*_at_ is the number of atoms in the unit cell and *N*_*k*_ is the number of *k* points used to discretize the Brillouin zone. To achieve the scaling reduction, it is required to use a spatially local basis instead of plane waves. The local basis can be chosen as a real-space grid where studies of unit cells of up to 100 atoms have been reported.^29,30^ Another choice of the spatially local basis is an atomic-orbital-like basis.^31^ This choice is highly efficient in the *GW* space–time method, enabling *GW* calculations on molecules with more than 1000 atoms.^32−36^

Periodic boundary conditions in the *GW* space–time method with atomic-orbital-like basis functions have not yet been reported. The main inhibiting factor has been the inclusion of *k*-dependent Coulomb interactions which represent a major challenge regarding computational efficiency and numerical precision.^37−39^ In this work, we overcome this challenge by employing real space representations of the polarizability, the screened Coulomb interaction, and the self-energy. The real-space representation allows us to use the minimum image convention (MIC)^40,41^ (i.e., each atomic orbital in the simulation interacts only with the closest image of another atomic orbital). We benchmark the algorithm on *G*_0_*W*_0_ band gaps of monolayer MoS_2_, MoSe_2_, WS_2_, and WSe_2_, finding an average deviation of only 0.06 eV from reference calculations.^42,43^ We also apply the *GW* algorithm to a MoSe_2_/WS_2_ bilayer with an unprecedented cell size of 984 atoms which has an order of magnitude more atoms than previous state-of-the-art large-scale *GW* calculations.^25^

## 2 Algorithm

The *GW* algorithm presented in this work starts from a density functional theory (DFT) calculation,
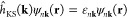
1where ε_*n***k**_ represents the eigenvalues of the Kohn–Sham Hamiltonian *ĥ*_KS_(**k**). Bloch orbitals ψ_*n***k**_ are expanded in Gaussians,^44^
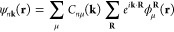
2where the molecular orbital coefficients *C*_*μn*_(**k**) are optimized in DFT and ϕ_μ_^**R**^(**r**) are Gaussian-type basis functions being centered on an atom in cell **R**.

Following the *GW* space–time method,^29^ we compute the Green’s function in imaginary time at the Γ point,^32^
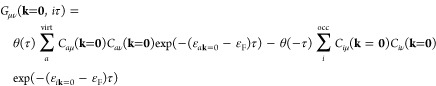
3The irreducible polarizability χ_*PQ*_(*iτ*) at **k** = **0** in the Gaussian auxiliary basis {φ_*P*_^**R**^(**r**)} follows,^35^
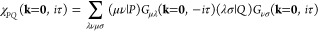
4using three-center matrix elements
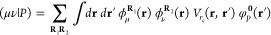
5of the truncated Coulomb operator
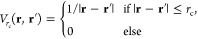
6with cutoff radius *r*_c_. The tensor (*μν*|*P*) can be understood as originating from the resolution of the identity with the truncated Coulomb metric (RI-tCm),^35,45,46^ where *r*_c_ is typically chosen to be 3 Å.^35,46,47^ The locality of *V*_*r*_c__(**r**, **r**′) ensures that the tensor (*μν*|*P*) is sparsely occupied, making the *GW* algorithm computationally efficient. RI-tCm ensures that the resolution of the identity quickly converges with the size of the auxiliary basis {φ_*P*_^**R**^(**r**)}.^35,45,46^ We use Tikhonov regularization^48^ for the RI expansion to prevent linear dependencies of fit coefficients, as we discuss in detail in the Supporting Information.

The polarizability χ_*PQ*_(**k**, *iτ*) is needed on a dense *k*-point mesh because it is later multiplied with the bare Coulomb interaction *V*(**k**) that diverges at the Γ point and thus requires a fine *k*-point sampling. The atom-centered basis allows us to decompose the Γ-point result (eq 4), χ_*PQ*_(**k** = **0**, *iτ*), using
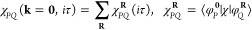
7where χ_*PQ*_^**R**^ is the real-space representation of the irreducible polarizability and φ_*P*_^**R**^ denotes an auxiliary Gaussian orbital which is localized in cell **R**. For nonmetallic systems, the polarizability χ(**r**, **r**′, *iτ*) is space-local (i.e., χ(**r**, **r**′, *iτ*) exponentially decays with increasing |**r** – **r**′|).^49,50^ The matrix element χ_*PQ*_^**R**^ thus vanishes in the case of a large distance between the center of φ_*P*_^**0**^ and the center of φ_*Q*_^**R**^. We employ MIC (i.e., we assume that χ_*PQ*_^**R**^(*iτ*) in eq 7 is nonzero only if the atomic center of φ_*P*_^**0**^ and the atomic center of φ_*Q*_^**R**^ are closest together among all cells **R**). In this way, we extract χ_*PQ*_^**R**^(*iτ*) from eq 7,
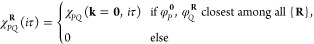
8which is exact in the limit of a large, nonmetallic unit cell. Using eq 8, we obtain the polarizability at any *k* point at negligible computational cost,
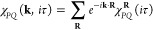
9We transform the irreducible polarizability to an imaginary frequency,^29,30,51^
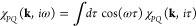
10We have observed that χ_*PQ*_(**k**, *iω*) computed from eq 10 features spurious negative eigenvalues with small absolute values. The reason is that eq 9 together with eq 8 is exact only in the limit of large unit cells. We remove negative eigenvalues from χ_*PQ*_(**k**, *iω*), which requires costly diagonalization for every *k* point and every frequency point ω. Once having a positive definite matrix χ_*PQ*_(**k**, *iω*), we compute the dielectric function
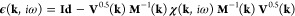
11where **Id** is the identity matrix and the truncated Coulomb matrix **M**(**k**) appears due to the RI-tCm,
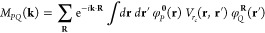
12We regularize **M**^–1^(**k**) to prevent linear dependencies in the RI expansion; see details in the Supporting Information.

**V**(**k**) in eq 11 denotes the Coulomb matrix^38,52^
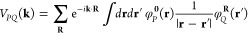
13The sum over **R** reaches over the entire crystal. Following the elaborate discussion in the Appendix of ref (52), we evaluate the lattice sum (eq 13) on a finite set of cells {**R**}. The subset fulfills ∑_{**R**}_*e*^–*i***k**·**R**^ = 0 for all **k** in our *k*-point mesh to ensure absolute convergence of the lattice sum (eq 13) for 2D periodic systems.^52^ Typically, we employ a few thousand neighbor cells to evaluate the lattice sum (eq 13) which is computationally affordable thanks to analytical Coulomb integrals.^53^

We decompose the screened Coulomb interaction *W* = *V* + (ϵ^–1^ – 1)*V* into the bare Coulomb interaction *V* and the correction (ϵ^–1^ – 1)*V* due to screening. The bare interaction leads to the exchange self-energy Σ^*x*^, and it is well known^54,55^ that truncating the bare interaction in Γ-only Hartree–Fock calculations leads to fast convergence in the supercell size. We therefore use the truncated Coulomb operator *V*_*r*_c_^HF^_ from eq 6 for the bare interaction,
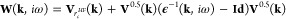
14For the cutoff radius *r*_c_^HF^ in eq 14, we set half the minimum of inner box wall distances in periodic directions, which is the common choice in periodic Hartree–Fock calculations.^55^

We transform *W* to real space,
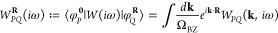
15where Ω_BZ_ is the Brillouin zone (BZ) volume. Special care is required for the BZ integral as *W*_*PQ*_(**k**, *iω*) diverges at the Γ point with 1/*k* for two-dimensional materials if φ_*P*_ and φ_*Q*_ are s-type basis functions.^37,38,56^ We evaluate *W*_*PQ*_(**k**, *iω*) using a 4 × 4 Monkhorst–Pack *k*-point mesh^57^ {**k**_*j*_}_*j*=1_^*N*_*k*_^, *N*_*k*_ = 8,^58^ and a 6 × 6 Monkhorst–Pack *k*-point mesh {**q**_*j*_}_*j*=1_^*N*_*q*_^, *N*_*q*_ = 18. We extrapolate the BZ integration (eq 15) with the inverse square root of the number of *k* points.^38,59^ In practice, we extrapolate with respect to *k* points by discretizing eq 15 to
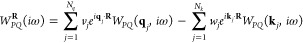
16where *k*-point extrapolation is absorbed in integration weights
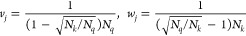
17

Following the *GW* space–time method,^29,30^ we compute the self-energy Σ(**r**, **r**′, *iτ*) = *iG*(**r**, **r**′, *iτ*) *W*(**r**, **r**′, *iτ*).^29^ Σ(**r**, **r**′, *iτ*) is space-local as *G*(**r**, **r**′,*iτ*) is space-local,^49^ and only elements of *W*(**r**, **r**′, *iτ*) with small |**r** – **r**′| contribute to Σ. We thus continue with the minimum image of eq 15
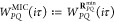
18where the cell vector
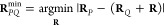
19gives the smallest distance between the atomic centers **R**_*P*_ of φ_*P*_^**0**^ and the atomic center **R**_*Q*_ + **R** of φ_*Q*_^**R**^. We include the Γ-point RI metric matrix **M** from eq 12,
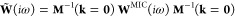
20which leads to the self-energy at the Γ point,
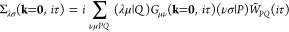
21*k* points in Σ follow from MIC at negligible computational cost (cf. eqs 8 and 9),
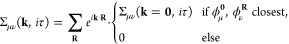
22We transform the self-energy to real energy^18,29,30,32,35^ and to the Bloch basis to compute quasiparticle energies ε_*n***k**_^*G*_0_*W*_0_^,
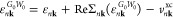
23where *v*_*n***k**_^xc^ is the diagonal of the exchange-correlation matrix.

## 3 Numerical Precision

**Figure 1:**
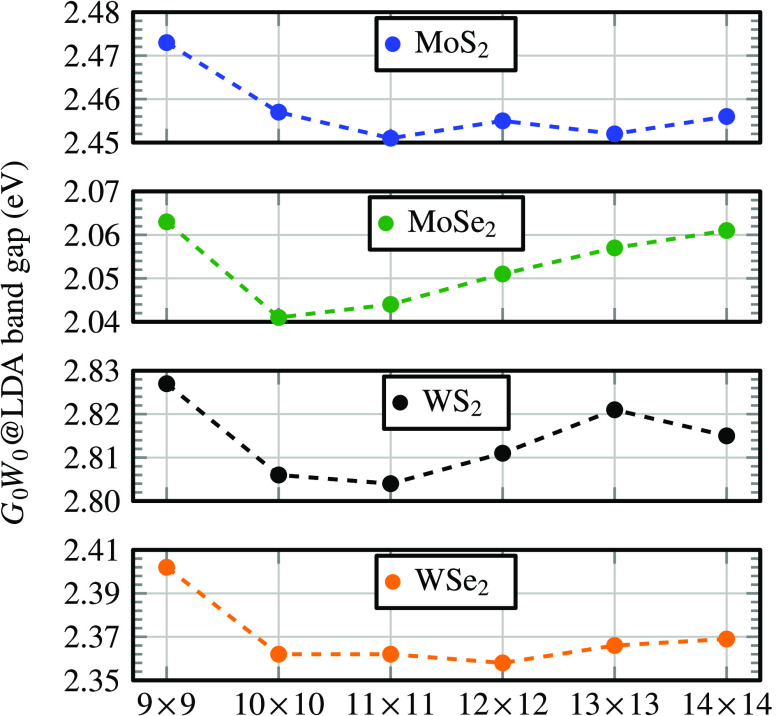
*G*_0_*W*_0_ band gap of monolayer WS_2_, MoS_2_, WSe_2_, and MoSe_2_ calculated from eq 23 as a function of the supercell size (TZVP-MOLOPT basis set,^60^ without spin–orbit coupling (SOC)).

The numerical trick in the presented *GW* algorithm is the MIC used in eqs 8, 18, and 22. MIC is exact in the limit of a large unit cell. We determine the critical cell size for the validity of MIC by computing the *G*_0_*W*_0_ band gap of monolayers MoS_2_, MoSe_2_, WS_2_, and WSe_2_, presented in Figure 1. For the four materials, the band gap changes on average by only 11 meV between the 10 × 10 supercell (300 atoms in the unit cell) and the 14 × 14 supercell (588 atoms in the unit cell). We conclude that the *GW* algorithm from this work can be used to study unit cells that are as large as a 10 × 10 supercell or larger. In the Supporting Information, we show additional convergence tests on the basis set size, the number of time and frequency points, the *k*-point mesh size, the filter threshold for sparse operations, and the vertical box height.

We compare the *G*_0_*W*_0_ band gap of monolayers MoS_2_, MoSe_2_, WS_2_, and WSe_2_ to the *G*_0_*W*_0_ band gap computed from three different plane-wave codes;^39,42,43^ see Table 1. We find that our *G*_0_*W*_0_ band gaps deviate on average by only 0.06 eV to the band gaps from plane-wave-based codes. This small discrepancy might be due to the use of different pseudopotentials and the difficulty in reaching the complete basis set limit.

**Table 1:** *G*_0_*W*_0_@PBE Band Gap (in eV, without SOC) of Monolayers MoS_2_, MoSe_2_, WS_2_, and WSe_2_ Computed from Equation 23 (TZV2P-MOLOPT Basis,^60^ 10 × 10 Supercell, Detailed Convergence Test in the SI) and Computed from Plane-Wave Codes^39,42,43^^a^

Software package	MoS_2_	MoSe_2_	WS_2_	WSe_2_
This work (CP2K^61^)	2.47	2.07	2.81	2.37
GPAW^42^	2.53	2.12	2.75	2.30
BerkeleyGW^39^	2.45	2.09	2.61	2.34
VASP^43^	2.50	2.06	2.70	2.34

a Details of BerkeleyGW calculations are given in the SI. We have removed SOC from the GPAW band gaps.^42^.

## 4 Computational Effort

**Figure 2:**
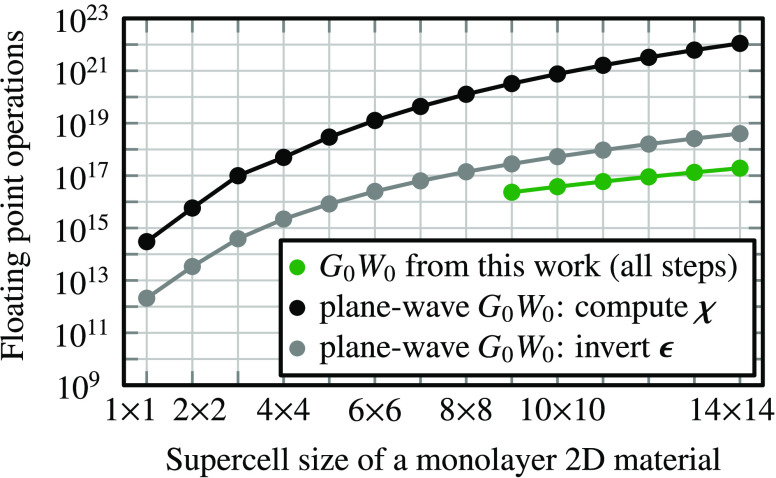
Number of floating point operations (real double precision) needed for executing *G*_0_*W*_0_ algorithms. Green: low-scaling *G*_0_*W*_0_ algorithm from this work using a TZVP-MOLOPT basis set.^60^ Black: computing the irreducible polarizability **χ** in a plane-wave basis. Gray: inverting the dielectric matrix **ϵ** in a plane-wave basis. Underlying computational parameters are typical for monolayers MoS_2_, MoSe_2_, WS_2_, and WSe_2_; see the detailed raw data and discussion available in the SI.

The presented *G*_0_*W*_0_ algorithm has a significantly reduced computational cost compared to plane-wave-based *G*_0_*W*_0_ algorithms. We show the number of floating point operations to compute the irreducible polarizability **χ** in a plane-wave *G*_0_*W*_0_ algorithm in Figure 2 in black. As a comparison, we show the execution of our low-scaling *G*_0_*W*_0_ algorithm via green traces. Our *G*_0_*W*_0_ algorithm requires 40,000 times fewer floating point operations for a 14 × 14 supercell as computing **χ** in plane waves for a 14 × 14 supercell. One might reduce the computational cost for computing χ in plane waves by a stochastic evaluation of the unoccupied band summation.^23^ For such an algorithm, the inversion of the dielectric matrix **ϵ** remains a computationally costly step. As we show in Figure 2, inverting **ϵ** in a plane-wave basis requires an order of magnitude more floating point operations compared to executing our whole *G*_0_*W*_0_ algorithm.

Further advantages compared to plane-wave-based algorithms include the cheap diagonalization of the Kohn–Sham matrix to obtain Bloch states thanks to the compact Gaussian basis. Also, nonperiodic directions are easily dealt with in our *GW* algorithm by restricting the sum over cells **R** to periodic directions. It is not necessary to truncate the Coulomb operator in nonperiodic directions as in plane-wave algorithms.^39,62^ Moreover, the self-energy (eq 22) is available in the full Gaussian basis set, which allows us to compute the *G*_0_*W*_0_ correction for all Bloch states at negligible computational cost.

**Figure 3:**
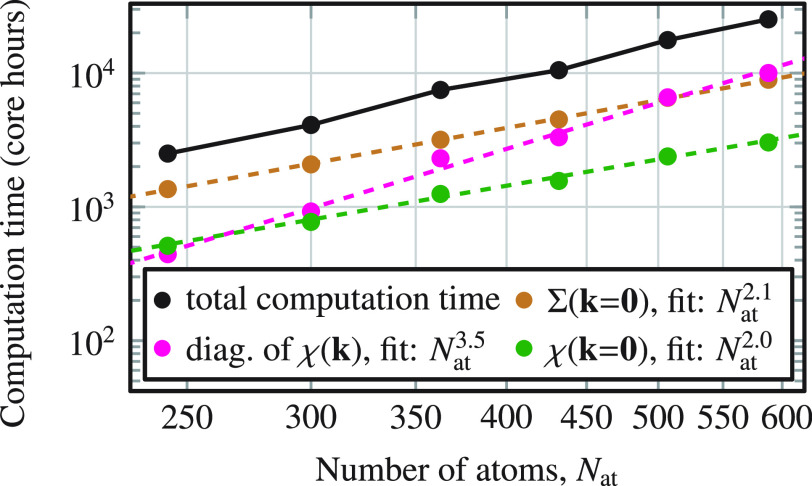
Execution time of a *G*_0_*W*_0_ calculation for MoSe_2_ 9 × 9–14 × 14 supercells (TZVP-MOLOPT basis set) on Supermuc-NG (Intel Skylake Xeon Platinum 8174). Magenta points show the computational cost to diagonalize the polarizability χ(**k**), which allows us to remove all spurious negative eigenvalues of χ(**k**) to ensure numerical stability. Dashed lines show a fit of *αN*_at_^β^ to the execution time, where α and β are fit parameters. Raw data are available in the SI.

We measured the computation time of the algorithm, shown in Figure 3. The computation time is moderate; as an example, a *G*_0_*W*_0_ calculation on the 10 × 10 MoSe_2_ supercell (300 atoms) takes only 7 h on 576 cores. Assuming ideal scalability starting from the 9 × 9 cell, we estimate that a *G*_0_*W*_0_ calculation on 4500 atoms is in reach.^63^ Scalability improvements are the subject of ongoing work to achieve this system size in practice.

## 5 Application: 2D Heterobilayer

We now focus on an application of the *G*_0_*W*_0_ algorithm to transition-metal dichalcogenide heterobilayers which recently gained increased attention due to twist-angle-dependent moiré potentials and interlayer excitons.^3,4,7−9,12−15^ Recent large-scale plane-wave-based *GW* calculations on twisted heterostructures were limited to 75 atoms in the unit cell.^25^ This *GW* computation^25^ has been described to be highly cumbersome, and it was achieved only by using an advanced accelerated large-scale version of the BerkeleyGW code which scales to entire leadership high-performance computers with more than half a million cores.^22,23^ Small unit cells with 75 atoms allow for only the study of heterobilayers with selected, large twist angles and absent atomic reconstruction.

**Figure 4:**
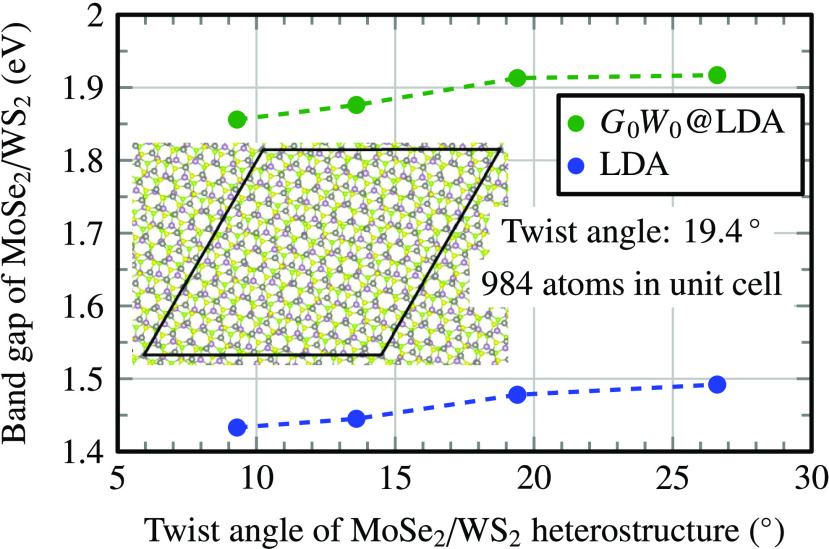
Band gap of a MoSe_2_/WS_2_ heterostructure as a function of the twist angle. Inset: The unit cell (black rhomboid) for a 19.4° twist angle contains 984 atoms.

In order to illustrate the large-scale capabilities of our *G*_0_*W*_0_ algorithm beyond monolayers, we focus on prototypical MoSe_2_/WS_2_ twisted heterostructures. On one hand, the different lattice parameters of MoSe_2_ and WS_2_ give rise to a considerably large moiré periodicity at zero twist angle (∼8 nm), thus requiring a large number of atoms in the structure. On the other hand, low-angle MoSe_2_/WS_2_ has shown an interesting interplay of intra- and interlayer exciton hybridization because of the nearly degenerate conduction bands. This feature, however, is still under debate in the literature.^3,7,13−15^ The underlying electronic structure is thus crucial to resolve this controversy and is exactly the kind of problem that requires large-scale *GW* calculations. Here we considered MoSe_2_/WS_2_ moiré superstructures with twist angles of between 9.3 and 26.6° (Figure 4) that have corresponding unit cells of up to 984 atoms. We emphasize that in all cases the strain of the individual monolayers is <0.01% compared to the experimentally determined lattice constants,^64,65^ which is important because the band gap is very sensitive to strain.^66,67^ The *G*_0_*W*_0_ band gap of the MoSe_2_/WS_2_ bilayer depends on the twist angle changing from 1.86 eV (9.3°) to 1.92 eV (26.8°), in line with experimental observations of the exciton emission energy.^13^ Our *GW* calculation on the 984-atom heterostructure takes 42 h on only 1536 cores, which is a factor of 30,000 faster than with a plane-wave algorithm; see the estimate in the SI. Such large-scale *GW* calculations are an ideal starting point for further analysis of the electronic structure of these materials. For example, with our *GW* algorithm, the calculation of deep moiré potentials^4^ is within reach because of atomic reconstruction and height variations. Both crucially influence the interlayer screening that is captured by the *GW* method. On top of a *GW* calculation, the Bethe-Salpeter equation^17,19^ will enable the study of excitons in large-scale moiré structures. Our computationally efficient scheme also holds great promise for nanoscale excited-state dynamics in low-dimensional materials with Green’s function methods.

## 6 Conclusions

We have presented a low-scaling *GW* algorithm with periodic boundary conditions employing localized basis functions and the minimum image convention. The *GW* algorithm is numerically precise and requires up to 5 orders of magnitude fewer floating point operations compared to plane-wave codes. We carried out a *G*_0_*W*_0_ calculation on a MoSe_2_/WS_2_ heterostructure with 984 atoms in the unit cell which is an order of magnitude more than the state of the art.^25^ Our *GW* algorithm will enable routine applications of *GW* and its time-dependent variants to low-dimensional, nanostructured materials that were previously computationally highly challenging.

## Data Availability

The low-scaling *GW* algorithm is implemented in the open-source CP2K package,^61^ which is freely available from GitHub.^68^ Inputs and outputs of the calculations are also available on GitHub.^69^

## Supporting Information Available

Further computational parameters underlying our calculations (Sec. S1); regularized resolution of the identity used in the *GW* algorithm (Sec. S2); numerical convergence of our benchmark monolayer band gaps (Sec. S3, S4); resolution of the identity with the truncated Coulomb metric in Sec. S5; periodic, low-scaling *GW* algorithm from the manuscript to a *GW* algorithm with standard scaling (Sec. S6); the number of required floating point operations in our low-scaling Gaussian algorithm, a standard-scaling Gaussian algorithm and in planewave algorithms (Sec. S7 – S11); timings of our algorithm (Sec. S12); comparison of the supercell convergence of our algorithm to the supercell convergence of stochastic *GW* (Sec. S13) (PDF)
