# Supplementary material for: Low-Scaling GW Algorithm Applied to Twisted Transition-Metal Dichalcogenide Heterobilayers
Source: J Chem Theory Comput. 2024 Feb 14;20(5):2202–8. doi: 10.1021/acs.jctc.3c01230 (PMC10938508; doi:10.1021/acs.jctc.3c01230)
Supplement: Supplementary file 1 — ct3c01230_si_001.pdf [file ct3c01230_si_001.pdf]

Supporting Information to
"Low-scaling *GW* algorithm applied to twisted transition-metal dichalcogenide heterobilayers"

Maximilian Graml,[1,2] Klaus Zollner,[1] Daniel Hernangómez-Pérez,[3] Paulo E. Faria Junior,[1] and Jan Wilhelm[1,2,*]

[1]*Institute of Theoretical Physics, University of Regensburg, 93053 Regensburg, Germany*
[2]*Regensburg Center for Ultrafast Nanoscopy (RUN), University of Regensburg, 93053 Regensburg, Germany*
[3]*Department of Molecular Chemistry and Materials Science, Weizmann Institute of Science, Rehovot 7610001, Israel*

(Dated: December 28, 2023)

We list further computational parameters underlying our calculations (Sec. S1). The regularized resolution of the identity used in the *GW* algorithm is defined (Sec. S2). We further show the numerical convergence of our benchmark monolayer band gaps (Sec. S3, S4). We discuss the resolution of the identity with the truncated Coulomb metric in Sec. S5. We compare the periodic, low-scaling *GW* algorithm from the manuscript to a *GW* algorithm with standard scaling (Sec. S6). We also report and discuss the number of required floating point operations in our low-scaling Gaussian algorithm, a standard-scaling Gaussian algorithm and in plane-wave algorithms (Sec. S7 – S11). Timings of our algorithm are reported in Sec. S12. We compare the supercell convergence of our algorithm to the supercell convergence of stochastic *GW* in Sec. S13.

S1. COMPUTATIONAL DETAILS

The algorithm is implemented in the CP2K software package[1,2]. Unless otherwise noted, we use the TZVP-MOLOPT basis set[1] together with Goedecker-Teter-Hutter pseudopotentials[3] (charge $q_{\text{Mo}} = q_{\text{W}} = +14e$ and $q_{\text{S}} = q_{\text{Se}} = +6e$). Exemplarily, the TZVP-MOLOPT basis set contains 35 basis functions per Mo atom ($4\times$s, $3\times$p, $3\times$d, $1\times$f) and 17 basis functions per S atom ($3\times$s, $3\times$p, $1\times$d). As auxiliary basis, we use the relevant exponents from the RI-def2-SVP basis set[4]. Inputs and outputs are available on github[5]. We use a regularized resolution of the identity with regularization parameter 10^{-2}, see Sec. S2 for details.

We use a 4×4 and 6×6 Monkhorst-Pack k-point mesh[6] when calculating $\chi(\mathbf{k})$, $\epsilon(\mathbf{k})$, $V(\mathbf{k})$, $V_{r_c}(\mathbf{k})$, $W(\mathbf{k})$. We use an 8×8 k-point mesh for the *GW* self-energy. We compute three-center integrals of the truncated Coulomb integrals using the libint library[7]. Two-center integrals are computed using solid harmonic Gaussians[8]. We use minimax grids[9,10] with ten grid points for the Fourier transform in imaginary time and imaginary frequency. We employ the dbcsr tensory library[11,12] to execute parallel sparse matrix-tensor operations when computing χ and Σ.

For the plane-wave code benchmarks, the DFT calculations were performed using the Quantum Espresso package[13], with the PBE exchange-correlation functional[14] and norm-conserving non-relativistic pseudopotentials[15]. A basis cut-off of 100 Ry was employed, and the self-consistent charge density was converged on a $30 \times 30 \times 1$ **k**-grid with a total energy accuracy of 10^{-9} Ry. To determine the quasi-particle band-gap, a one-shot *GW* calculation (G_0W_0) was conducted using the BerkeleyGW package[16,17]. The Quantum Espresso DFT converged energies and Kohn-Sham states were used as the starting point. The dielectric matrix was computed with a dielectric cut-off of 25 Ry, considering a total of 1999 occupied and empty bands on a $12 \times 12 \times 1$ uniform **k**-grid. For the explicit full-frequency calculation, we employed the contour-deformation method with the Adler-Wiser formula. In order to accelerate convergence near the Γ-point ($|\mathbf{q}| \to 0$), a non-uniform neck subsampling approach was also considered[18]. The spurious interactions between periodic replicas in the perpendicular direction to the surface were removed with a Coulomb interaction truncation scheme[19].

The heterobilayers were created with the `CellMatch` code[20], implementing the coincidence lattice method[21,22].

S2. REGULARIZED RESOLUTION OF THE IDENTITY

In this section, we describe the regularization method in the resolution of the identity (RI) that we use in the *GW* algorithm. We show that the regularization accelerates the convergence of the *GW* bandgap with the supercell size.

RI starts from four-center Coulomb integrals (4c-CI)[23],

$$(\phi_\mu\phi_\nu|\phi_\lambda\phi_\sigma) := \int d\mathbf{r}\, d\mathbf{r}'\, \phi_\mu(\mathbf{r})\, \phi_\nu(\mathbf{r})\, \frac{1}{|\mathbf{r}-\mathbf{r}'|}\, \phi_\lambda(\mathbf{r}')\, \phi_\sigma(\mathbf{r}')\,, \tag{S1}$$

where ϕ_μ, ϕ_ν, ϕ_λ and ϕ_σ are atomic-orbital (AO) Gaussian basis functions. In RI, one expands the Gaussian products $\phi_\mu(\mathbf{r})\phi_\nu(\mathbf{r})$

and $\phi_\lambda(\mathbf{r}')\phi_\sigma(\mathbf{r}')$ using an auxiliary basis $\{\varphi_P(\mathbf{r})\}$,

$$\phi_\mu(\mathbf{r})\phi_\nu(\mathbf{r}) = \sum_P B^P_{\mu\nu}\,\varphi_P(\mathbf{r})\,. \tag{S2}$$

$B^P_{\mu\nu}$ are expansion coefficients that will be chosen to approximate $\phi_\mu(\mathbf{r})\phi_\nu(\mathbf{r})$. RI is used together with a metric $m(\mathbf{r},\mathbf{r}')$,[23–25] for example the Coulomb metric, $m(\mathbf{r},\mathbf{r}') = 1/|\mathbf{r}-\mathbf{r}'|$ or the overlap metric $m(\mathbf{r},\mathbf{r}') = \delta(|\mathbf{r}-\mathbf{r}'|)$. The expansion coefficients $B^P_{\mu\nu}$ depend on $m(\mathbf{r},\mathbf{r}')$ and are chosen, such that the following expression gets minimal:[23]

$$\left(\phi_\mu\phi_\nu - \sum_P B^P_{\mu\nu}\,\varphi_P\middle|\phi_\lambda\phi_\sigma - \sum_P B^P_{\lambda\sigma}\,\varphi_P\right)_m := \int d\mathbf{r}\,d\mathbf{r}'\left[\phi_\mu(\mathbf{r})\phi_\nu(\mathbf{r}) - \sum_P B^P_{\mu\nu}\,\varphi_P(\mathbf{r})\right] m(\mathbf{r},\mathbf{r}')\left[\phi_\lambda(\mathbf{r}')\phi_\sigma(\mathbf{r}') - \sum_P B^P_{\lambda\sigma}\,\varphi_P(\mathbf{r}')\right] \tag{S3}$$

$$= (\phi_\mu\phi_\nu|\phi_\lambda\phi_\sigma)_m - 2\sum_P B^P_{\mu\nu}(\phi_\mu\phi_\nu|\varphi_P)_m + \sum_{PQ} B^P_{\mu\nu}B^Q_{\lambda\sigma}M_{PQ}\,. \tag{S4}$$

The two- and three center integrals appearing in Eq. (S4) are defined as

$$(\phi_\mu\phi_\nu|\varphi_P)_m \equiv (\varphi_P|\phi_\mu\phi_\nu)_m = \int d\mathbf{r}\,d\mathbf{r}'\;\phi_\mu(\mathbf{r})\phi_\nu(\mathbf{r})\;m(\mathbf{r},\mathbf{r}')\;\varphi_P(\mathbf{r}')\,, \tag{S5}$$

$$M_{PQ} \equiv (\varphi_P|\varphi_Q)_m = \int d\mathbf{r}\,d\mathbf{r}'\;\varphi_P(\mathbf{r})\;m(\mathbf{r},\mathbf{r}')\;\varphi_Q(\mathbf{r}')\,. \tag{S6}$$

Differentiating Eq. (S4) with respect to $B^P_{\mu\nu}$ and setting the result to zero leads to the RI expansion coefficients,[23]

$$B^P_{\mu\nu} = \sum_Q \left(\mathbf{M}^{-1}\right)_{PQ}(\phi_\mu\phi_\nu|\varphi_Q)_m\,. \tag{S7}$$

$\mathbf{M}^{-1}$ is the inverse of matrix $\mathbf{M}$ that is defined in Eq. (S6). Inserting Eq. (S7) into Eqs. (S2)/(S1), one obtains the equation that is used to replace four-center Coulomb integrals by two-center and three-center integrals,

$$(\phi_\mu\phi_\nu|\phi_\lambda\phi_\sigma) = \sum_{PQRT}(\phi_\mu\phi_\nu|\varphi_P)_m\left(\mathbf{M}^{-1}\right)_{PQ}V_{QR}\left(\mathbf{M}^{-1}\right)_{RT}(\varphi_T|\phi_\lambda\phi_\sigma)_m\,, \tag{S8}$$

where the two-center Coulomb matrix is

$$V_{PQ} = \int d\mathbf{r}\,d\mathbf{r}'\;\varphi_P(\mathbf{r})\;\frac{1}{|\mathbf{r}-\mathbf{r}'|}\;\varphi_Q(\mathbf{r}')\,. \tag{S9}$$

In practical calculations, the RI basis $\{\varphi_P\}$ is large. This leads to linear dependencies in the RI basis which results in large inverse matrix elements M^{-1}_{PQ} and thus large expansion coefficients $B^P_{\mu\nu}$. In this work, we regularize Eq. (S3)/(S4), i.e. we minimize

$$\left(\phi_\mu\phi_\nu - \sum_P B^P_{\mu\nu}\,\varphi_P\middle|\phi_\lambda\phi_\sigma - \sum_P B^P_{\lambda\sigma}\,\varphi_P\right)_m + \alpha^2\sum_P (B^P_{\mu\nu})^2\,, \tag{S10}$$

where $\alpha \geq 0$. Differentiating Eq. (S10) with respect to $B^P_{\mu\nu}$ and setting the result to zero leads to

$$B^P_{\mu\nu} = \sum_Q \left((\mathbf{M}+\alpha\,\mathbf{Id})^{-1}\right)_{PQ}(\phi_\mu\phi_\nu|\varphi_Q)_m\,, \tag{S11}$$

where $\mathbf{Id}$ is the identity matrix.

In the algorithm described in the manuscript, we incorporate the regularized RI (S11), by inverting the matrix $\mathbf{M}(\mathbf{k}) + \alpha\mathbf{Id}$ to calculate the matrix denoted as $\mathbf{M}^{-1}(\mathbf{k})$,

$$\mathbf{M}^{-1}(\mathbf{k}) := \left(\mathbf{M}(\mathbf{k}) + \alpha\mathbf{Id}\right)^{-1}\,. \tag{S12}$$

We chose $\alpha = 10^{-2}$ for all calculations in the manuscript. In Fig. S1, we show that the G_0W_0 band gap of MoS_2 converges relatively fast with the supercell size when choosing $\alpha = 10^{-2}$ (blue traces) compared to $\alpha = 10^{-3}$ (green traces) and to standard RI ($\alpha = 0$, brown traces). An even larger regularization $\alpha = 10^{-1}$ hampers the RI expansion (S11); we observe that the G_0W_0@LDA band gap deviates by 0.03 eV for large cells compared to the G_0W_0@LDA band gap computed with smaller regularization parameters α.

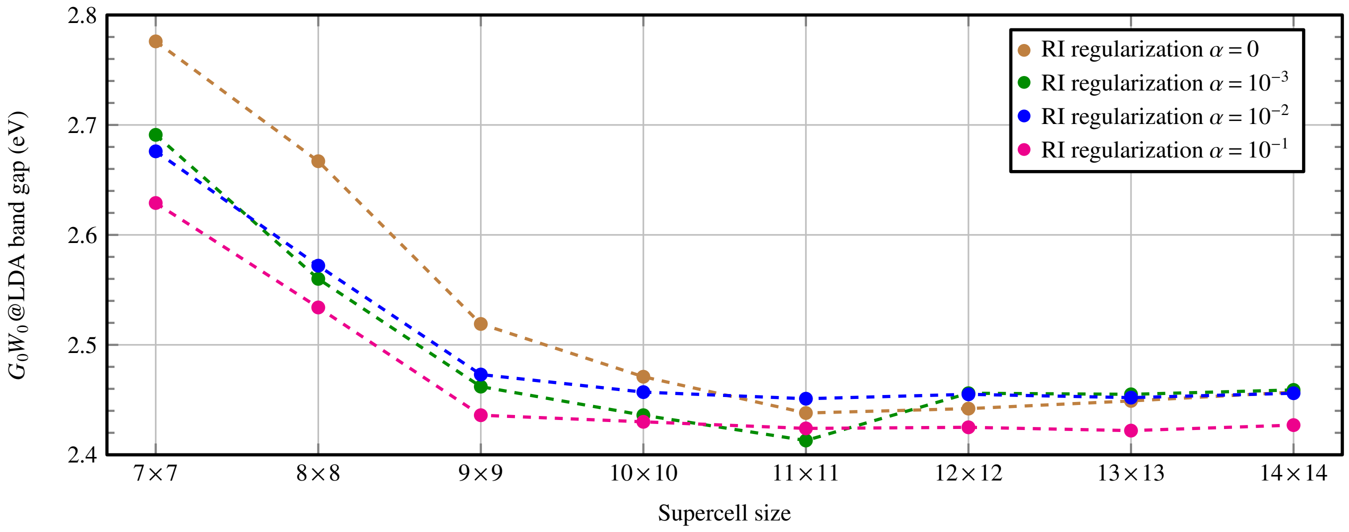

FIG. S1. G_0W_0@LDA gap for MoS_2 as function of the supercell size for three values of the RI regularization parameter $\alpha \in \{0, 10^{-3}, 10^{-2}, 10^{-1}\}$ [α enters the GW algorithm via Eq. (S12)].

S3. CONVERGENCE WITH THE BASIS SET SIZE

We investigate how the G_0W_0@PBE bandgap of monolayer MoS_2, $MoSe_2$, WS_2 and WSe_2 converges with the basis set $\{\phi_\nu\}$ for Bloch states and the auxiliary basis set $\{\varphi_P\}$ from the resolution of the identity (RI), see Table S1. We also change the supercell size and the number of time and frequency points[25]. For Table I in the manuscript, we report the bold G_0W_0@PBE bandgap (10×10 supercell, 30 time/frequency points, TZV2P-MOLOPT basis set). Further investigating and improving the numerical convergence with the basis set size, supercell size and the time/frequency grid, will be subject of future work.

TABLE S1. G_0W_0@PBE bandgap of a monolayer MoS_2, $MoSe_2$, WS_2, and WSe_2 for various different numerical parameters. We abbreviate "RI standard" for a standard RI basis set (example: 159 RI basis functions per MoS_2 unit), "RI big" (225 RI basis functions per MoS_2 unit) and "RI huge" (316 RI basis functions per MoS_2 unit). The TZVP-MOLOPT and the TZV2P-MOLOPT basis sets are standard basis sets[1,26] used together with Goedecker-Teter-Hutter pseudopotentials[3]. As an example, the TZVP-MOLOPT-GTH basis set contains 35 Gaussians per Mo atom (4 × s, 3 × p, 3 × d, 1 × f functions) and 17 Gaussians per S atom (3 × s, 3 × p, 1 × d functions). Inputs and outputs are available on github[5].

			Gaussian basis: TZVP-MOLOPT[1,26]			Gaussian basis: TZV2P-MOLOPT[1,26]		
	Supercell	Time/freq. grid points	RI standard	RI big	RI huge	RI standard	RI big	RI huge
MoS_2	10×10	10	2.459	2.538	2.510	2.455	2.560	2.511
	11×11	10	2.451	2.483		2.446	2.493	
	12×12	10	2.456	2.462		2.451		
	10×10	20	2.493			2.476		
	10×10	30	2.486			**2.471**		
$MoSe_2$	10×10	10	2.045	2.029	2.047	2.053	2.043	2.054
	11×11	10	2.047	2.051		2.054	2.058	
	10×10	20	2.079			2.074		
	10×10	30	2.077			**2.071**		
WS_2	10×10	10	2.796	2.786	2.787	2.793	2.782	2.771
	11×11	10	2.798	2.786		2.788	2.784	
	10×10	20	2.800			2.814		
	10×10	30	2.814			**2.812**		
WSe_2	10×10	10	2.365	2.350	2.355	2.347	2.333	2.336
	11×11	10	2.368	2.354		2.344	2.338	
	10×10	20	2.381			2.362		
	10×10	30	2.378			**2.367**		

S4. ADDITIONAL CONVERGENCE CHECKS

In Fig. S2, we present additional convergence checks of the GW bandgap with the minimax time/frequency grid size, the k-point mesh size, the filter parameter of sparse tensor operations, and with the box height.

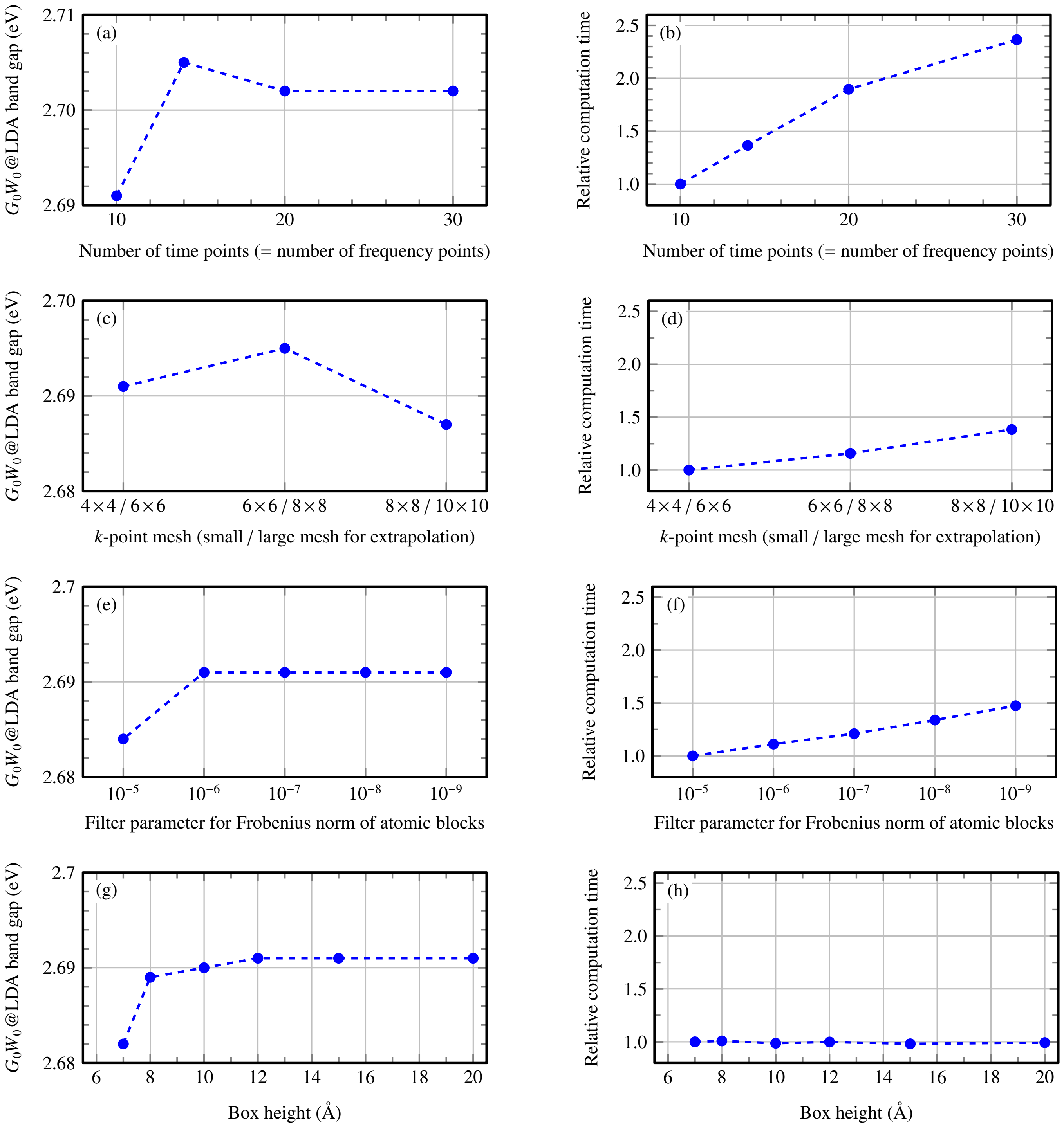

FIG. S2. Convergence tests of the low-scaling GW algorithm for a 7×7 supercell of MoS_2. We benchmark the convergence of the G_0W_0 gap as function of the following convergence parameters: (a)/(b) number of time/frequency points, (c)/(d) k-point mesh size, (e)/(f) filter parameter of sparse tensor operations and (g)/(h) box height. Shown are the bandgap (left column) and the computation time (right column). Standard settings in the manuscript are 10 time/frequency points, a 4×4 / 6×6 k-point mesh, a filter 10^{-6} and 15 Å box height.

S5. CUTOFF RADIUS OF THE TRUNCATED COULOMB METRIC FOR THE RESOLUTION OF THE IDENTITY

The choice of the RI metric $m(\mathbf{r},\mathbf{r}')$ is crucial for making the low-scaling GW algorithm from the manuscript efficient. One possibility is the overlap metric[23,25,27],

$$m(\mathbf{r},\mathbf{r}') = \delta(\mathbf{r}-\mathbf{r}') \tag{S13}$$

where δ is the Dirac distribution. The overlap metric is space-local in the sense that the auxiliary Gaussian basis functions φ_P do not overlap with atomic-orbital basis function products $\phi_\mu\phi_\nu$ in Eq. (S5) if there is enough distance between their centers. This leads to vanishing three-center overlap matrix elements $(\mu\nu|P)_m$ and increasing computational efficiency due to sparsity.

In contrast, the Coulomb metric

$$m_\mathrm{C}(\mathbf{r},\mathbf{r}') = \frac{1}{|\mathbf{r}-\mathbf{r}'|} \tag{S14}$$

couples RI basis functions φ_P and Gaussians basis function pairs $\phi_\mu\phi_\nu$ in Eq. (S5) over effectively infinite distances due to the slow polynomial decay of $1/|\mathbf{r}-\mathbf{r}'|$. With the Coulomb metric, no sparsity can be gained hampering its usage in low-scaling GW algorithms. In standard $O(N^4)$ algorithms, each Gaussian ϕ_μ, ϕ_ν is transformed to the delocalized molecular orbital basis $\{\psi_n\}$ loosing all sparsity anyway[28]. In such a conventional algorithm, where sparsity cannot be exploited, the Coulomb metric is the optimal choice because the RI factorization given in Eq. (S8) converges very quickly with respect to the RI basis set size.[23] The Coulomb metric thus yields generally higher accuracy than the overlap metric.

In this work, we employ the truncated Coulomb metric[24,25,29]

$$m_{r_\mathrm{c}}(\mathbf{r},\mathbf{r}') = \begin{cases} \dfrac{1}{|\mathbf{r}-\mathbf{r}'|} & \text{if } |\mathbf{r}-\mathbf{r}'| < r_\mathrm{c}\,, \\[2ex] 0 & \text{else}\,, \end{cases} \tag{S15}$$

where the Coulomb interaction is cut after a distance r_c. In the limit of a large cutoff radius r_c, the truncated Coulomb metric turns into the Coulomb metric, $\lim_{r_\mathrm{c}\to\infty} m_{r_\mathrm{c}}(\mathbf{r},\mathbf{r}') = 1/|\mathbf{r}-\mathbf{r}'|$. For a small cutoff radius r_c, calculations based on the truncated Coulomb metric are equivalent to calculations based on the overlap metric. The truncated Coulomb metric combines the attractive features of the Coulomb metric and the overlap metric: high accuracy due to the near-sighted Coulomb operator and preservation of sparsity due to the locality of $m_{r_\mathrm{c}}(\mathbf{r},\mathbf{r}')$. The RI factorization in Eq. (S8) is exact in the limit of a complete RI basis, independent of the chosen RI metric. Therefore, truncating the Coulomb operator with a finite r_c does not affect the accuracy of the GW algorithm as long as the RI basis is sufficiently large.

We show the convergence of the G_0W_0 bandgap of MoS_2 with the RI basis set size $\{\varphi_P(\mathbf{r})\}$ in Fig. S3. It is seen that the convergence with the RI basis is fast when using a cutoff radius a cutoff radius $r_\mathrm{c} = 3$ Å, and the convergence is slower for $r_\mathrm{c} = 2$ Å. For a large RI basis set with 240 auxiliary functions per MoS_2 unit, we find that the bandgap for $r_\mathrm{c} = 2$ Å matches the bandgap for $r_\mathrm{c} = 3$ Å within 0.02 eV . Overall, we confirm that $r_\mathrm{c} = 3$ Å is a good choice.

We note that in plane-wave implementations, RI with different metrics is not discussed. The reason is that the Coulomb matrix, the truncated Coulomb matrix and the overlap matrix are diagonal in the plane-wave basis. As consequence, RI factorizations as in Eq. (S8) are identical for the three different metrics when using plane wave basis functions. More details can be found in the Supporting Information of Ref. 25.

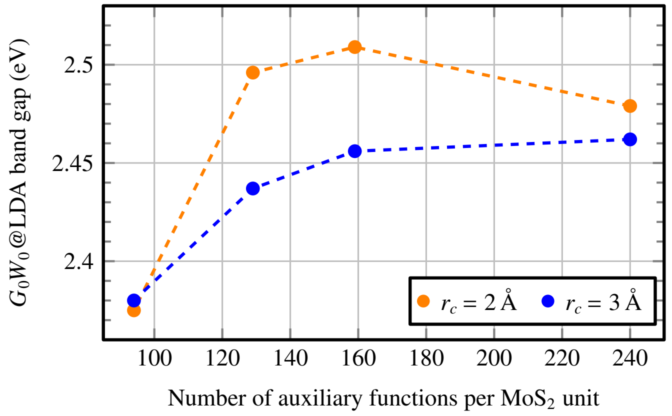

FIG. S3. Gap of monolayer MoS_2 (12×12 supercell) as function of the number of auxiliary functions $\{\varphi_P\}$ and the cutoff radius in RI metric.

S6. COMPARISON OF THE LOW-SCALING *GW* ALGORITHM TO A STANDARD-SCALING *GW* ALGORITHM

In Fig. S4, we compare the periodic, Gaussian-based, low-scaling *GW* algorithm from the manuscript with a periodic, Gaussian-based *GW* algorithm that operates in frequency only and avoids sparse matrix-tensor operations.

Periodic *GW* algorithm in a Gaussian basis with RI (similar to the algorithm by Zhu and Chan[28])	Low-scaling periodic *GW* algorithm in a Gaussian basis with RI (this work)
Irreducible polarizability (scaling: $N_k^2 N_\text{occ} N_\text{virt} N_\text{RI}^2$)<br>$\chi_{PQ}(\mathbf{k},i\omega) = \frac{1}{N_k}\sum_{ia\mathbf{q}} (i\mathbf{k}\,a\mathbf{k{-}q}\mid P\mathbf{q})\,\frac{2(\varepsilon_{i\mathbf{k}}-\varepsilon_{a\mathbf{k-q}})}{\omega^2+(\varepsilon_{i\mathbf{k}}-\varepsilon_{a\mathbf{k-q}})^2}\,(i\mathbf{k}\,a\mathbf{k{-}q}\mid Q\mathbf{q})$<br>$(i\mathbf{k}\,a\mathbf{k{-}q}\mid P\mathbf{q})$ are three-center integrals of the RI metric $m(\mathbf{r},\mathbf{r}')$, see Ref. 28 for a definition.	Irreducible polarizability<br>1. Γ-point only (scaling for large systems[25]: N^2):<br>$\chi_{PQ}(\mathbf{0},i\tau) = \sum_{\lambda\nu\mu\sigma} (\mu\mathbf{0}\,\nu\mathbf{0}\mid P\mathbf{0})\,G_{\mu\lambda}(\mathbf{0},-i\tau)\,(\lambda\mathbf{0}\,\sigma\mathbf{0}\mid Q\mathbf{0})\,G_{\nu\sigma}(\mathbf{0},i\tau)$<br>2. Γ-point to arbitrary k-point:<br>$\chi_{PQ}(\mathbf{k},i\tau) = \sum_{\mathbf{R}} e^{i\mathbf{k}\cdot\mathbf{R}}\cdot\begin{cases}\chi_{PQ}(\mathbf{0},i\tau) & \text{if } \varphi_P^{\mathbf{0}},\varphi_Q^{\mathbf{R}} \text{ closest,}\\ 0 & \text{else}\end{cases}$<br>3. Time to frequency transform:<br>$\chi_{PQ}(\mathbf{k},i\omega) = \int d\tau\,\cos(\omega\tau)\,\chi_{PQ}(\mathbf{k},i\tau)$<br>4. Remove all spurious negative eigenvalues of $\chi_{PQ}(\mathbf{k},i\omega)$
Dielectric function (scaling: $N_k N_\text{RI}^3$)<br>$\boldsymbol{\epsilon}(\mathbf{k},i\omega) = \mathbf{1} - \mathbf{V}^{0.5}(\mathbf{k})\mathbf{M}^{-1}(\mathbf{k})\boldsymbol{\chi}(\mathbf{k},i\omega)\mathbf{M}^{-1}(\mathbf{k})\mathbf{V}^{0.5}(\mathbf{k})$	Dielectric function (scaling: $N_k N_\text{RI}^3$)<br>$\boldsymbol{\epsilon}(\mathbf{k},i\omega) = \mathbf{1} - \mathbf{V}^{0.5}(\mathbf{k})\mathbf{M}^{-1}(\mathbf{k})\boldsymbol{\chi}(\mathbf{k},i\omega)\mathbf{M}^{-1}(\mathbf{k})\mathbf{V}^{0.5}(\mathbf{k})$
Screened Coulomb potential (scaling: $N_k N_\text{RI}^3$)<br>$\mathbf{W}(\mathbf{k},i\omega) = \mathbf{V}_{r_c}(\mathbf{k}) + \mathbf{V}^{0.5}(\mathbf{k})\left(\boldsymbol{\epsilon}^{-1}(\mathbf{k},i\omega)-\mathbf{Id}\right)\mathbf{V}^{0.5}(\mathbf{k})$<br>$\tilde{\mathbf{W}}(\mathbf{k},i\omega) = \mathbf{M}^{-1}(\mathbf{k})\,\mathbf{W}(\mathbf{k},i\omega)\,\mathbf{M}^{-1}(\mathbf{k})$	Screened Coulomb potential (scaling: $N_k N_\text{RI}^3$)<br>$\mathbf{W}(\mathbf{k},i\omega) = \mathbf{V}_{r_c}(\mathbf{k}) + \mathbf{V}^{0.5}(\mathbf{k})\left(\boldsymbol{\epsilon}^{-1}(\mathbf{k},i\omega)-\mathbf{Id}\right)\mathbf{V}^{0.5}(\mathbf{k})$<br>$\mathbf{W}^{\mathbf{R}}(i\omega) = \frac{1}{N_k}\sum_{\mathbf{k}} e^{i\mathbf{k}\cdot\mathbf{R}}\,\mathbf{W}(\mathbf{k},i\omega)$<br>$W_{PQ}^\text{MIC}(i\omega) = W_{PQ}^{\mathbf{R}_{PQ}^\text{min}}(i\omega)\,,\quad \mathbf{R}_{PQ}^\text{min} = \underset{\mathbf{R}}{\text{argmin}}\left\lvert\mathbf{R}_P-(\mathbf{R}_Q+\mathbf{R})\right\rvert$<br>$\tilde{\mathbf{W}}(i\omega) = \mathbf{M}^{-1}(\mathbf{0})\,\mathbf{W}^\text{MIC}(i\omega)\,\mathbf{M}^{-1}(\mathbf{0})$
Self-energy (scaling: $N_k^2 N_\text{AO}^2 N_\text{RI}^2$)<br>$\Sigma_{n\mathbf{k}}(i\omega) = -\frac{1}{2\pi N_k}\sum_{m\mathbf{q}}\int d\omega'\,\frac{1}{i(\omega-\omega')+\varepsilon_\text{F}-\varepsilon_{m\mathbf{k-q}}}$<br>$\times\sum_{PQ}(n\mathbf{k}\,m\mathbf{k{-}q}\mid P\mathbf{q})\,\tilde{W}_{PQ}(\mathbf{q},i\omega')\,(n\mathbf{k}\,m\mathbf{k{-}q}\mid Q\mathbf{q})$	Self-energy<br>1. Γ-point only (scaling for large systems[25]: N^2)<br>$\Sigma_{\lambda\sigma}(\mathbf{0},i\tau) = i\sum_{\nu\mu PQ}(\lambda\mathbf{0}\,\mu\mathbf{0}\mid Q\mathbf{0})\,G_{\mu\nu}(\mathbf{0},i\tau)\,(\nu\mathbf{0}\,\sigma\mathbf{0}\mid P\mathbf{0})\,\tilde{W}_{PQ}(i\tau)$<br>2. Transformation to Bloch states (Escaling: $N_k N_\text{AO}^3$)<br>$\Sigma_{\lambda\sigma}(\mathbf{k},i\tau) = \sum_{\mathbf{R}} e^{i\mathbf{k}\cdot\mathbf{R}}\cdot\begin{cases}\Sigma_{\lambda\sigma}(\mathbf{0},i\tau) & \text{if } \phi_\lambda^{\mathbf{0}},\phi_\sigma^{\mathbf{R}} \text{ closest,}\\ 0 & \text{else}\end{cases}$<br>$\Sigma_{n\mathbf{k}}(i\tau) = \sum_{\lambda\sigma} C_{n\lambda}^*(\mathbf{k})\,\Sigma_{\lambda\sigma}(\mathbf{k},i\tau)\,C_{n\sigma}(\mathbf{k})$

FIG. S4. Left column: Periodic *GW* algorithm with Gaussian basis functions, similar to Ref. 28. Right column: Periodic *GW* algorithm with Gaussian basis functions from this work.

S7. REQUIRED NUMBER OF FLOATING POINT OPERATIONS OF A PLANE-WAVE BASED *GW* ALGORITHM

The computational effort of applying plane-wave based *GW* algorithms to two-dimensional materials is large. In a brute-force plane-wave implementation[17,30], the computational bottleneck is computing the irreducible polarizability,

$$\chi_{\mathbf{G}\mathbf{G}'}(\mathbf{q},i\omega) = \sum_n^{\text{occ}}\sum_{n'}^{\text{empty}}\sum_{\mathbf{k}} \frac{\langle n\mathbf{k}{+}\mathbf{q}|e^{i(\mathbf{q}+\mathbf{G})\cdot\mathbf{r}}|n'\mathbf{k}\rangle\,\langle n'\mathbf{k}|e^{-i(\mathbf{q}+\mathbf{G}')\cdot\mathbf{r}}|n\mathbf{k}{+}\mathbf{q}\rangle}{\varepsilon_{n\mathbf{k}+\mathbf{q}}-\varepsilon_{n'\mathbf{k}}+i\omega}\,, \qquad N_{\text{flop}}^{\text{PW}}(\chi) = 4N_{\mathbf{q}}N_\omega N_{\mathbf{G}}^2 N_{\text{occ}}N_{\text{empty}}N_{\mathbf{k}}\,, \tag{S16}$$

where $\mathbf{G},\mathbf{G}'$ are reciprocal lattice vectors, $\mathbf{q}$ is a vector in the first Brillouin zone, n,n' refer to occupied and empty bands, respectively, and the brackets in the second line denote integrals of a plane wave and Bloch states. $N_{\text{flop}}^{\text{PW}}(\chi)$ in Eq. (S16) denotes the number of floating point operations (real double precision) which is the product of:

- a factor 4 to account for the complex numbers,
- the number of $\mathbf{q}$-points, $N_{\mathbf{q}}$, to discretize $\chi(\mathbf{q})$ in the Brillouin zone, (a 3×3 mesh has been chosen in Ref. 31 for a large-scale calculation on a 2D material; considering time-reversal symmetry $\mathbf{q}\leftrightarrow-\mathbf{q}$, a 3×3 mesh contains 5 independent $\mathbf{q}$-points; the 3×3 mesh is necessary to resolve the divergence of the Coulomb interaction at $\mathbf{q}\to\mathbf{0}$)
- the number of frequency points, N_ω, (We assume full-frequency calculations[32] with $N_\omega = 10$ as in the main manuscript. When using a plasmon-pole model[31,32], one would have $N_\omega = 1$.)
- the number of plane waves, $N_{\mathbf{G}}^2$, where the square is due to the row and column index of χ, (From the energy cut-off $E_c = 25\,\text{Ry}$[31,32], we estimate the plane-wave cutoff $G_c = \sqrt{2m_eE_c}/\hbar$ and the corresponding reciprocal sphere volume $\Omega_c = 4\pi G_c^3/3$. From the 1×1 supercell size $V_{\text{cell}} = 2.5\,\text{Å}\times2.5\,\text{Å}\times25\,\text{Å}$of a 2D semiconductor, we estimate the Brillouin zone volume $\Omega_{\text{BZ}} = (2\pi)^3/V_{\text{cell}}$ which leads to $N_{\mathbf{G}} = \Omega_c/\Omega_{\text{BZ}} = 2200$ for a 1×1 supercell.)
- the number of occupied bands, N_{occ}, (which follows from the employed pseudopotential)
- the number of empty bands, N_{empty}, (which is a convergence parameter and has been chosen in Ref. 32 as 6000 for a 1×1 cell, we assume a factor 10 less empty bands for large-scale calculations) and
- the number of $\mathbf{k}$-points, $N_{\mathbf{k}}$, for the Brillouin zone sum $\sum_{\mathbf{k}}$ (for large-scale calculations, the Γ-point is sufficient, i.e. $N_{\mathbf{k}} = 1$).

Another computationally demanding operation in plane-wave *GW* algorithms is to invert the dielectric matrix $\epsilon_{\mathbf{G}\mathbf{G}'}(\mathbf{q},i\omega)$,[17]

$$\epsilon^{-1}_{\mathbf{G}\mathbf{G}'}(\mathbf{q},i\omega) := (\epsilon(\mathbf{q},i\omega))^{-1}_{\mathbf{G}\mathbf{G}'}\,, \qquad N_{\text{flop}}^{\text{PW}}(\epsilon) = N_{\mathbf{q}}N_\omega N_{\mathbf{G}}^3\,. \tag{S17}$$

We estimate the required floating point operations to evaluate Eq. (S16) and (S17) in Table S2. In case of stochastic sampling[30] of the occupied-virtual sum in Eq. (S16), the computational prefactor and the system size scaling of Eq. (S16) may be reduced. The inversion of ϵ in Eq. (S17) needs to be executed independently of stochastic subsampling and requires an order of magnitude more FLOPs compared to the low-scaling *GW* algorithm from this work (see Table S3).

TABLE S2. We estimate the required operations to compute the irreducible polarizability in a plane-wave basis set, Eq. (S16), for a 2D semiconductor as monolayer MoS_2. Computational parameters are chosen similar to Refs. 31 and 32.

Supercell	N_{occ}	N_{empty}	$N_{\mathbf{G}}$	$N_{\mathbf{q}}$	$N_{\mathbf{k}}$	N_ω	$N_{\text{flop}}^{\text{PW}}(\chi)$	$N_{\text{flop}}^{\text{PW}}(\epsilon)$
1x1	13	600	2200	20	10	10	$3.0\cdot10^{14}$	$2.1\cdot10^{12}$
2x2	52	2400	8800	5	3	10	$5.8\cdot10^{15}$	$3.4\cdot10^{13}$
3x3	117	5400	19800	5	2	10	$9.9\cdot10^{16}$	$3.9\cdot10^{14}$
4x4	208	9600	35200	5	1	10	$4.9\cdot10^{17}$	$2.2\cdot10^{15}$
6x6	468	21600	79200	5	1	10	$1.3\cdot10^{19}$	$2.5\cdot10^{16}$
8x8	832	38400	140800	5	1	10	$1.3\cdot10^{20}$	$1.4\cdot10^{17}$
10x10	1300	60000	220000	5	1	10	$7.6\cdot10^{20}$	$5.3\cdot10^{17}$
12x12	1872	86400	316800	5	1	10	$3.2\cdot10^{21}$	$1.6\cdot10^{18}$
14x14	2548	117600	431200	5	1	10	$1.1\cdot10^{22}$	$4.0\cdot10^{18}$

S8. REQUIRED NUMBER OF FLOATING POINT OPERATIONS OF THE *GW* ALGORITHM FROM THIS WORK

We compute χ in this work in three steps[25],

$$\chi_{PQ}(\mathbf{q}{=}\mathbf{0}, i\tau) = \sum_{\lambda\nu} X_{\lambda\nu P}(i\tau)\, Y_{\lambda\nu Q}(i\tau)\,, \qquad N_{\text{flop}} = N_\tau N_{\text{RI}}^2 N_{\text{AO}}^2\, \alpha\,, \tag{S18}$$

$$X_{\lambda\nu P}(i\tau) = \sum_{\mu} (\mu\nu|P)\, G_{\mu\lambda}(\mathbf{k}{=}\mathbf{0}, -i\tau)\,, \qquad Y_{\lambda\nu Q}(i\tau) = \sum_{\sigma} (\lambda\sigma|Q)\, G_{\nu\sigma}(\mathbf{k}{=}\mathbf{0}, i\tau)\,, \qquad N_{\text{flop}} = 2N_\tau N_{\text{RI}} N_{\text{AO}}^3\, \alpha\,. \tag{S19}$$

The required floating point operations, N_{flop}, is computed as a product of:

- the number of time points, N_τ, [cf. Fig. S2 (a)]
- the number of auxiliary RI basis functions, N_{RI},
- the number of Gaussian basis functions N_{AO} to expand Bloch states,
- α is the percentage of matrix elements $(\mu\nu|P)$ above a certain filter threshold [cf. Fig. S2 (c)]. All matrix elements smaller than the threshold are neglected in the computation. For a large number of atoms N_{at} in the unit cell, α is smaller than one,

$$\alpha \sim \left(\frac{N_{\text{at}}^*}{N_{\text{at}}}\right)^2\,, \tag{S20}$$

  where N_{at}^* is the number of atoms of the largest system without sparsity, i.e. $\alpha = 1$ for a system with N_{at}^* atoms. With Eq. (S20), the computational scaling of Eqs. (S18) and (S19) is $O(N_{\text{at}}^2)$ for $N_{\text{at}} > N_{\text{at}}^*$.

The number of auxiliary Gaussians is typically two to three times larger than the basis set for Bloch orbitals, $N_{\text{RI}} \approx 2\text{-}3{\cdot}N_{\text{AO}}$. This results in a similar number of floating point operations of Eq. (S18) and (S19). Computing the self-energy is expected to require a similar number of floating point operations as Eq. (S18) and (S19). The complex diagonalization of $\chi_{PQ}(\mathbf{k}, i\omega)$ to remove spurious negative eigenvalues is estimated to require $10N_{\text{RI}}^3 N_{\mathbf{k}} N_\omega$ operations, where $N_\omega = N_\tau$.[25] We choose a $4{\times}4$ and 6×6 k-mesh to extrapolate the Brillouin zone integration of W. This extrapolation requires $N_{\mathbf{k}} = 26$ kpoints when considering time-reversal symmetry $\mathbf{k} \leftrightarrow -\mathbf{k}$.

Summarizing, we estimate the total required number of floating point operations as

$$N_{\text{flop}}^{\text{Gauss}} = 4N_\tau N_{\text{RI}}^2 N_{\text{AO}}^2\, \alpha + 10N_{\text{RI}}^3 N_{\mathbf{k}} N_\omega\,. \tag{S21}$$

Results for $N_{\text{flop}}^{\text{Gauss}}$ for monolayer 2D semiconductors are summarized in Table S3.

TABLE S3. Required number of operations (S21) to execute the G_0W_0 algorithm from this work for a 2D semiconductor as monolayer MoS_2.

Supercell	N_{AO}	N_{RI}	$N_\tau = N_\omega$	α	$N_{\mathbf{k}}$	$N_{\text{flop}}^{\text{Gauss}}$
1x1	69	159		1.000		
9x9	5589	12879	10	0.160	26	$3.9 \cdot 10^{16}$
10x10	6900	15900	10	0.105	26	$6.1 \cdot 10^{16}$
11x11	8349	19239	10	0.072	26	$9.3 \cdot 10^{16}$
12x12	9936	22896	10	0.051	26	$1.4 \cdot 10^{17}$
13x13	11661	26871	10	0.037	26	$1.9 \cdot 10^{17}$
14x14	13524	31164	10	0.027	26	$2.7 \cdot 10^{17}$

S9. COMPARISON OF FLOATING POINT OPERATIONS OF *GW* ALGORITHM FROM THIS WORK TO BRUTE-FORCE PLANE-WAVE *GW* ALGORITHM

The presented GW algorithm has several computational advantages over plane-wave-based GW algorithms. The computational bottleneck in plane-wave-based GW algorithms is the calculation of the irreducible polarizability from Eq. (S16). The required number of floating point of this operation is given in Table S2. The required number of operations of our presented G_0W_0 algorithm is given in Table S3. Our G_0W_0 algorithm requires a similar number of floating point operations for a 9×9 supercell as a plane-wave G_0W_0 algorithm for a 3×3 supercell. For a 14×14 supercell, our G_0W_0 algorithm requires 40,000 times less operations compared to a plane-wave based algorithm. This large factor has several origins, most important are the following: The plane-wave basis $\{e^{i\mathbf{G}\cdot\mathbf{r}}\}$ for χ resolves large vacuum regions[31,32] for two-dimensional materials and is thus a factor 10 larger than the Gaussian auxiliary basis $\{\varphi_P\}$. We thus need to calculate 100 times less matrix elements of χ in a Gaussian basis compared to Eq. (S16). Three-center integrals over Gaussians are sparse due to the spatial locality of Gaussians[25]. Only 3 % of the integrals need to be considered for a 14×14 supercell reducing the number of operations by another factor 30. In the present algorithm, χ is evaluated at the Γ-point using real-valued matrix algebra[25] which makes another factor 4 compared to the complex matrix algebra in Eq. (S16). In Eq. (S16), at least a 3×3 mesh for $\mathbf{q}$ is necessary[31] which is responsible for another factor of 5[33]. These numerical parameters thus explain a factor of 60,000 between the required operations of a plane-wave G_0W_0 algorithm and the G_0W_0 algorithm from this work.

S10. REQUIRED NUMBER OF FLOATING POINT OPERATIONS OF A STANDARD-SCALING *GW* ALGORITHM WITH GAUSSIAN BASIS FUNCTIONS

In a standard-scaling GW algorithm, χ is computed in imaginary time with the Adler-Wiser (AW) formula[34,35],

$$\chi_{PQ}(\mathbf{q}{=}\mathbf{0}, i\omega) = 2\sum_i^{\text{occ}}\sum_a^{\text{empty}} \frac{\varepsilon_{i\mathbf{k}=0}-\varepsilon_{a\mathbf{k}=0}}{\omega^2+(\varepsilon_{i\mathbf{k}=0}-\varepsilon_{a\mathbf{k}=0})^2}\,, \qquad N_{\text{flop}}^{\text{AW}} = N_\omega N_{\text{RI}}^2 N_{\text{occ}} N_{\text{virt}}\,. \tag{S22}$$

The required floating point operations, N_{flop}, is computed as a product of:

- the number of frequency points, N_ω, typically $N_\omega = 10$ when using a minimax grid[9],
- the number of auxiliary RI basis functions, N_{RI}, typically $N_{\text{RI}} \approx 2\text{-}3\cdot N_{\text{AO}}$, where N_{AO} is the number of Gaussian basis functions to expand Bloch states,
- the number of occupied states, N_{occ},
- the number of empty states, N_{virt}, which is determined by the Gaussian basis set as we have $N_{\text{AO}} = N_{\text{occ}} + N_{\text{virt}}$,[36]

Similarly to Eq. (S21) we need to diagonalize $\chi_{PQ}(\mathbf{k}, i\omega)$ to remove spurious negative eigenvalues leading to the total required number of floating point operations

$$N_{\text{flop}}^{\text{Gauss,AW}} = N_\omega N_{\text{RI}}^2 N_{\text{occ}} N_{\text{virt}} + 10 N_{\text{RI}}^3 N_{\mathbf{k}} N_\omega\,. \tag{S23}$$

Results for $N_{\text{flop}}^{\text{Gauss,AW}}$ for monolayer 2D semiconductors are summarized in Table S4. For a 9x9 cell, the number of floating point operations is lower than the low-scaling space-time[37] GW algorithm (Table S3). For a 14x14 cell, the low-scaling space-time algorithm (S18), (S19) requires a similar number of operations compared to using the Adler-Wiser formula (S22). For larger systems, the low-scaling space-time algorithm will be computationally more efficient than the Adler-Wiser formula (S22).

TABLE S4. Required number of operations (S23) to execute the standard-scaling G_0W_0 algorithm with the Adler-Wiser formula (S22) for a 2D semiconductor as monolayer MoS_2.

Supercell	N_{occ}	N_{virt}	N_{RI}	N_ω	$N_{\mathbf{k}}$	$N_{\text{flop}}^{\text{Gauss,AW}}$
1x1	13	56	159			
9x9	1053	4536	12879	10	26	$1.35\cdot10^{16}$
10x10	1300	5600	15900	10	26	$2.89\cdot10^{16}$
11x11	1573	6776	19239	10	26	$5.80\cdot10^{16}$
12x12	1872	8064	22896	10	26	$1.10\cdot10^{17}$
13x13	2197	9464	26871	10	26	$2.01\cdot10^{17}$
14x14	2548	10976	31164	10	26	$3.50\cdot10^{17}$

S11. FLOATING POINT OPERATIONS FOR THE LARGEST $MOSE_2/WS_2$ HETEROSTRUCTURE WITH 984 ATOMS

We evaluate Eqs. (S16) and (S21) to compute the required number of floating point operations for the 984-atom $MoSe_2/WS_2$ heterostructure shown in Fig. 4 in the manuscript. For the large-scale Gaussian-based algorithm from this work, we have the computational parameters (defined in Sec. S8) and corresponding operations $N_{\text{flop}}^{\text{Gauss}}$ [Eq. (S21)]:

$$N_\tau = N_\omega = 10\,, \qquad N_{\text{RI}} = 49441\,, \qquad N_{\text{AO}} = 22632\,, \qquad \alpha = 0.66\,\%\,, \qquad N_{\mathbf{k}} = 26\,, \qquad N_{\text{flop}}^{\text{Gauss}} = 6.5\cdot 10^{17}\,. \tag{S24}$$

For a plane-wave G_0W_0 algorithm, the relevant computational parameters and corresponding operations $N_{\text{flop}}^{\text{PW}}(\chi)$ [Eq. (S16)] are

$$N_\omega = 10\,, \quad N_{\text{occ}} = 4264\,, \quad N_{\text{empty}} = 196800\,, \quad N_{\mathbf{G}} = 320000\,, \quad N_{\mathbf{q}} = 5\,, \quad N_{\mathbf{k}} = 1\,, \quad N_{\text{flop}}^{\text{PW}}(\chi) = 1.7\cdot 10^{22}\,. \tag{S25}$$

S12. TIMINGS OF THE *GW* ALGORITHM

TABLE S5. Timings of G_0W_0@LDA calculations on monolayer $MoSe_2$ supercells of different size on Supermuc-NG (processor type: Intel Skylake Xeon Platinum 8174, 48 cores per node, 768 GB memory per node).

Supercell size	Number of nodes	Total time (h)	Time of Eq. (S18)/(S19) (h)	Time of diag. of $\chi_{PQ}(i\omega,\mathbf{k})$ (h)	Time for Σ (h)
9×9	6	8.7	1.8	1.5	4.7
10×10	12	7.1	1.3	1.6	3.6
11×11	20	7.8	1.3	2.4	3.3
12×12	22	10.0	1.5	3.1	4.3
13×13	28	13.1	1.8	4.9	4.9
14×14	34	15.5	1.9	6.1	5.5

S13. SUPERCELL SIZE CONVERGENCE OF THE PRESENT *GW* ALGORITHM COMPARED TO STOCHASTIC *GW*

In this section, we discuss the convergence of *GW* gaps with respect to the simulation cell size. Our algorithm converges for supercells that are larger than the characteristic decay length of polarizability and Green's functions. Stochastic *GW*[38] also relies on large supercells that need to be larger than the characteristic electron-electron correlation length.

The supercell convergence of the stochastic G_0W_0 gap has been tested for monolayer and bilayer phosphorene in Ref 38, see Fig. S5 (a). For monolayer phosphorene (blue traces), the size convergence is slow. The G_0W_0 gap of the two largest calculations ($1/L = 0.0076\,a_0^{-1}$ and $1/L = 0.0098\,a_0^{-1}$) differ by 0.12 eV. An extrapolation towards infinitely large supercells is necessary which yields a G_0W_0 gap of 2.07 eV, in good agreement with reference calculations on phosphorene[38]. The slope of the extrapolation line deviates from the slope taking only the two largest calculations by $\sim 50\,\%$ which might be a sign of numerical instabilities of large-scale calculations. The convergence of bilayer phosphorene (green traces) seems faster than for the monolayer because the two largest points differ by only 0.02 eV. A confirmation of the bilayer convergence with an even larger supercell size has not been reported in Ref. 38.

In Figure S5 (b), we show the convergence of the G_0W_0 gap with respect to the supercell size for monolayer phosphorene calculated by our low-scaling *GW* algorithm. The G_0W_0 gap of the five largest supercells agree within only 14 meV. In comparison, the stochastic G_0W_0 gaps of phosphorene change from the supercell with $1/L = 0.015\,a_0^{-1}$ to the largest supercell with $1/L = 0.0076\,a_0^{-1}$ by 250 meV (monolayer) and 150 meV (bilayer). We conclude that our algorithm is superior in supercell size convergence over the stochastic *GW* algorithm presented in Ref. 38.

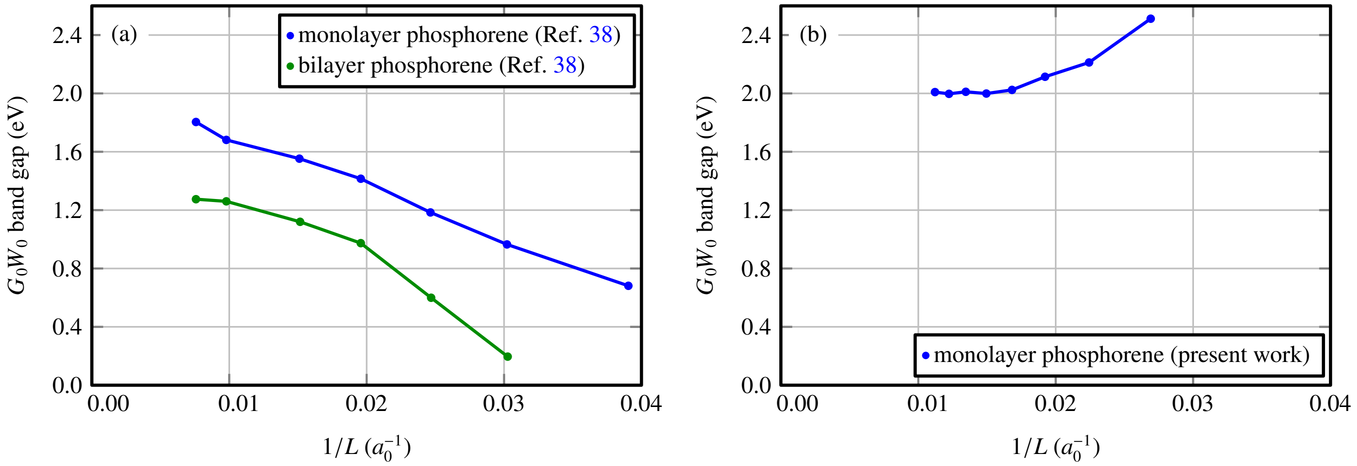

FIG. S5. Convergence of the G_0W_0 band gap with respect to the inverse characteristic length L of two-dimensional materials in units of the Bohr radius a_0. (a) Supercell convergence of stochastic-G_0W_0 gaps for monolayer phosphorene and bilayer phosphorene from Ref. 38. (b) Supercell convergence of low-scaling-G_0W_0 gaps from this work for monolayer phosphorene.

---

* jan.wilhelm@physik.uni-regensburg.de

[1] T. D. Kühne, M. Iannuzzi, M. Del Ben, V. V. Rybkin, P. Seewald, F. Stein, T. Laino, R. Z. Khaliullin, O. Schütt, F. Schiffmann, D. Golze, J. Wilhelm, S. Chulkov, M. H. Bani-Hashemian, V. Weber, U. Borštnik, M. Taillefumier, A. S. Jakobovits, A. Lazzaro, H. Pabst, T. Müller, R. Schade, M. Guidon, S. Andermatt, N. Holmberg, G. K. Schenter, A. Hehn, A. Bussy, F. Belleflamme, G. Tabacchi, A. Glöß, M. Lass, I. Bethune, C. J. Mundy, C. Plessl, M. Watkins, J. VandeVondele, M. Krack, and J. Hutter, J. Chem. Phys. **152**, 194103 (2020).

[2] `https://github.com/cp2k/cp2k`, accessed December 20, 2023.

[3] S. Goedecker, M. Teter, and J. Hutter, Phys. Rev. B **54**, 1703 (1996).

[4] F. Weigend, M. Häser, H. Patzelt, and R. Ahlrichs, Chem. Phys. Lett. **294**, 143 (1998).

[5] `https://github.com/JWilhelm/Inputs_outputs_low_scaling_GW_TMDC`, accessed December 20, 2023.

[6] H. J. Monkhorst and J. D. Pack, Phys. Rev. B **13**, 5188 (1976).

[7] Libint, A library for the evaluation of molecular integrals of operators over Gaussian functions, accessed December 20, 2023, "https://github.com/evaleev/libint," .

[8] D. Golze, N. Benedikter, M. Iannuzzi, J. Wilhelm, and J. Hutter, J. Chem. Phys. **146**, 034105 (2017).

[9] P. Liu, M. Kaltak, J. Klimeš, and G. Kresse, Phys. Rev. B **94**, 165109 (2016).

[10] M. Azizi, J. Wilhelm, D. Golze, M. Giantomassi, R. L. Panadés-Barrueta, F. A. Delesma, A. Buccheri, A. Gulans, P. Rinke, C. Draxl, and X. Gonze, J. Open Source Softw. **8**, 5570 (2023).

[11] J. Wilhelm, P. Seewald, M. Del Ben, and J. Hutter, J. Chem. Theory Comput. **12**, 5851 (2016).

[12] P. Seewald, *Low-Scaling Electronic Structure Methods Based on Sparse Tensor Contraction*, Ph.D. thesis, University of Zurich (2021).

[13] P. Giannozzi, O. Andreussi, T. Brumme, O. Bunau, M. B. Nardelli, M. Calandra, R. Car, C. Cavazzoni, D. Ceresoli, M. Cococcioni, N. Colonna, I. Carnimeo, A. D. Corso, S. de Gironcoli, P. Delugas, R. A. DiStasio, A. Ferretti, A. Floris, G. Fratesi, G. Fugallo, R. Gebauer, U. Gerstmann, F. Giustino, T. Gorni, J. Jia, M. Kawamura, H.-Y. Ko, A. Kokalj, E. Küçükbenli, M. Lazzeri, M. Marsili, N. Marzari, F. Mauri, N. L. Nguyen, H.-V. Nguyen, A. O. de-la Roza, L. Paulatto, S. Poncé, D. Rocca, R. Sabatini, B. Santra, M. Schlipf, A. P. Seitsonen, A. Smogunov, I. Timrov, T. Thonhauser, P. Umari, N. Vast, X. Wu, and S. Baroni, J. Phys. Condens. Matter **29**, 465901 (2017).

[14] J. P. Perdew, K. Burke, and M. Ernzerhof, Phys. Rev. Lett. **77**, 3865 (1996).

[15] M. J. van Setten, M. Giantomassi, E. Bousquet, M. J. Verstraete, D. R. Hamann, X. Gonze, and G.-M. Rignanese, Comput. Phys. Commun. **226**, 39 (2018).

[16] M. S. Hybertsen and S. G. Louie, Phys. Rev. B **34**, 5390 (1986).

[17] J. Deslippe, G. Samsonidze, D. A. Strubbe, M. Jain, M. L. Cohen, and S. G. Louie, Comput. Phys. Commun. **183**, 1269 (2012).

[18] F. H. da Jornada, D. Y. Qiu, and S. G. Louie, Phys. Rev. B **95**, 035109 (2017).

[19] S. Ismail-Beigi, Phys. Rev. B **73**, 233103 (2006).

[20] P. Lazic, Comput. Phys. Commun. **197**, 324 (2015).

[21] D. S. Koda, F. Bechstedt, M. Marques, and L. K. Teles, J. Phys. Chem. C **120**, 10895 (2016).

[22] S. Carr, S. Fang, and E. Kaxiras, Nat. Rev. Mater. **5**, 748 (2020).

[23] O. Vahtras, J. Almlöf, and M. Feyereisen, Chem. Phys. Lett. **213**, 514 (1993).

[24] Y. Jung, A. Sodt, P. M. W. Gill, and M. Head-Gordon, Proc. Natl. Acad. Sci. U.S.A. **102**, 6692 (2005).

[25] J. Wilhelm, P. Seewald, and D. Golze, J. Chem. Theory Comput. **17**, 1662 (2021).

[26] J. VandeVondele and J. Hutter, J. Chem. Phys. **127**, 114105 (2007).

[27] J. Wilhelm, D. Golze, L. Talirz, J. Hutter, and C. A. Pignedoli, J. Phys. Chem. Lett. **9**, 306 (2018).
[28] T. Zhu and G. K.-L. Chan, J. Chem. Theory Comput. **17**, 727 (2021).
[29] A. Luenser, H. F. Schurkus, and C. Ochsenfeld, J. Chem. Theory Comput. **13**, 1647 (2017).
[30] M. Del Ben, F. H. da Jornada, A. Canning, N. Wichmann, K. Raman, R. Sasanka, C. Yang, S. G. Louie, and J. Deslippe, Comput. Phys. Commun. **235**, 187 (2019).
[31] S. Kundu, T. Amit, H. R. Krishnamurthy, M. Jain, and S. Refaely-Abramson, arXiv: 2209.05030 (2022).
[32] D. Y. Qiu, F. H. da Jornada, and S. G. Louie, Phys. Rev. B **93**, 235435 (2016).
[33] Considering time-reversal symmetry, only 5 irreducible k-points are contained in a 3×3 mesh.
[34] S. L. Adler, Phys. Rev. **126**, 413 (1962).
[35] N. Wiser, Phys. Rev. **129**, 62 (1963).
[36] Remark: The Gaussian basis is rather compact, so all empty states that are available in the Gaussian basis set are included in Eq. (S22). This is in contrast to plane-wave calculations where the number of unoccupied states is a convergence parameter. The corresponding convergence parameter in calculations with a Gaussian basis set is the Gaussian basis set size, e.g. going from TZVP-MOLOPT to TZV2P-MOLOP, see Table S1.
[37] H. N. Rojas, R. W. Godby, and R. J. Needs, Phys. Rev. Lett. **74**, 1827 (1995).
[38] J. Brooks, G. Weng, S. Taylor, and V. Vlček, J. Phys. Condens. Matter **32**, 234001 (2020).
